# 1-[5-(3,4-Dichlorophenyl)-3-(2-naphthyl)-4,5-dihydropyrazol-1-yl]ethanone

**DOI:** 10.1107/S1600536808026858

**Published:** 2008-08-23

**Authors:** Zhi-Ke Lu, Shen Li, Yan Feng

**Affiliations:** a Forestry College, GuangXi University, Nanning 530005, People’s Republic of China; b Chemistry Department, Guangxi Industrial Vocational Technical College, Nanning 530001, People’s Republic of China

## Abstract

In the title compound, C_21_H_16_Cl_2_N_2_O, the central pyrazoline ring makes dihedral angles of 90.1 (3) and 7.8 (3)°, with the pendant benzene ring and naphthalene ring system, respectively. In the crystal structure, weak C—H⋯O inter­actions lead to chains of mol­ecules.

## Related literature

For related literature, see: Lu *et al.* (2006 ▶).
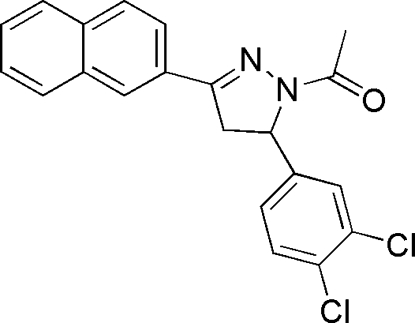


## Experimental

### 

#### Crystal data

C_21_H_16_Cl_2_N_2_O
*M* *_r_* = 383.26
Triclinic, 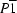

*a* = 6.2154 (12) Å
*b* = 9.3505 (19) Å
*c* = 16.319 (3) Å
α = 97.42 (3)°
β = 99.07 (3)°
γ = 104.12 (3)°
*V* = 894.2 (3) Å^3^
*Z* = 2
Mo *K*α radiation
μ = 0.38 mm^−1^
*T* = 113 (2) K
0.22 × 0.20 × 0.12 mm

#### Data collection

Rigaku Saturn diffractometer
Absorption correction: multi-scan (*CrystalClear*; Rigaku, 2004 ▶) *T*_min_ = 0.922, *T*_max_ = 0.956
9131 measured reflections
3155 independent reflections
2709 reflections with *I* > 2σ(*I*)
*R*_int_ = 0.037

#### Refinement

*R*[*F* ^2^ > 2σ(*F* ^2^)] = 0.033
*wR*(*F* ^2^) = 0.090
*S* = 1.04
3155 reflections
236 parameters
H-atom parameters constrained
Δρ_max_ = 0.20 e Å^−3^
Δρ_min_ = −0.28 e Å^−3^

### 

Data collection: *CrystalClear* (Rigaku, 2004 ▶); cell refinement: *CrystalClear*; data reduction: *CrystalClear*; program(s) used to solve structure: *SHELXS97* (Sheldrick, 2008 ▶); program(s) used to refine structure: *SHELXL97* (Sheldrick, 2008 ▶); molecular graphics: *SHELXTL* (Sheldrick, 2008 ▶); software used to prepare material for publication: *SHELXTL*.

## Supplementary Material

Crystal structure: contains datablocks global, I. DOI: 10.1107/S1600536808026858/hb2778sup1.cif

Structure factors: contains datablocks I. DOI: 10.1107/S1600536808026858/hb2778Isup2.hkl

Additional supplementary materials:  crystallographic information; 3D view; checkCIF report

## Figures and Tables

**Table 1:** Hydrogen-bond geometry (Å, °)

*D*—H⋯*A*	*D*—H	H⋯*A*	*D*⋯*A*	*D*—H⋯*A*
C6—H6⋯O1^i^	0.93	2.58	3.501 (2)	171

Symmetry code: (i) 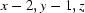.

**Fig. 1.**
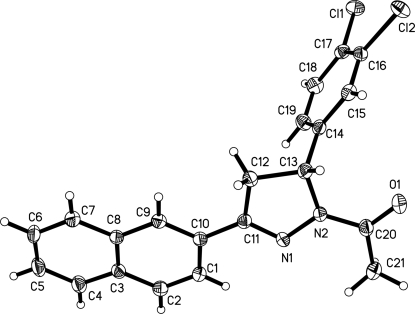
The molecular structure of (I), shown with 30% probability displacement ellipsoids (arbitrary spheres for H atoms).

**Fig. 2.**
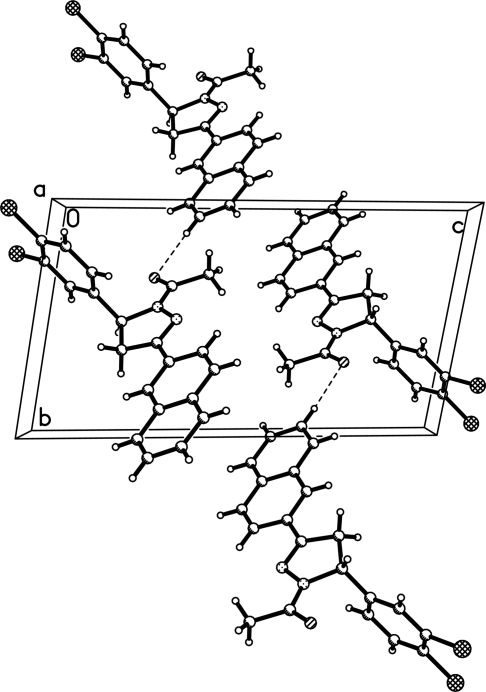
Part of the crystal structure of (I) showing C—H···O interactions as dashed lines.

## supplementary crystallographic information

### Comment

The title compound, (I), (Fig. 1) was prepared and structurally characterized as part of our ongoing studies (Lu *et al.*, 2006) of pyrazoline derivatives.

The pendant C14—C19 benzene ring and C1—C10 naphthalene ring make dihedral angles of 90.1 (3) and 7.8 (3)°, respectively, with the central N1/N2/C11/C12/C13 pyrazoline ring.  The dihedral angle between the C14—C19 ring and the C1—C10 ring is 86.8 (3)°.  The molecule of (I) is chiral: in the arbitrarily chosen asymmetric unit, C13 has *R* configuration, but crystal symmetry generates a racemic mixture.  In the crystal of (I), molecules are linked by a weak C—H···O interaction (Table 1 and Fig. 2) into infinite chains.

### Experimental

A mixture of 1-(naphthalen-2-yl)-3-(3,4-dichlorophenyl)prop-2-en-1-one (5.0 mmol), hydrazine hydrate (25.0 mmol) and acetic acid (30 ml) was heated at reflux for 5 h, then poured onto crushed ice. The precipitate was separated by filtration, washed with petroleum ether, and crystallized from ethyl acetate-petroleum ether to obtain the title compound.

The title compound (40 mg) was dissolved in a mixture of ethyl acetate (10 ml) and petroleum ether (30 ml) and the solution was kept at room temperature for 8 d. Natural evaporation of the solution gave colourless slabs of (I) suitable for X-Ray analysis. *M*.p. 515–516 K.

### Refinement

All the H atoms were placed geometrically (C—H = 0.93–0.98 Å), and refined as riding with *U*_iso_(H) = 1.2*U*_eq_(C) or 1.5*U*_eq_(methyl C).

### Crystal data

**Table:**

C_21_H_16_Cl_2_N_2_O	*Z* = 2
*M**_r_* = 383.26	*F*_000_ = 396
Triclinic, *P*1	*D*_x_ = 1.423 Mg m^−^^3^
Hall symbol: -P 1	Melting point: 515-516 K K
*a* = 6.2154 (12) Å	Mo *K*α radiation λ = 0.71073 Å
*b* = 9.3505 (19) Å	Cell parameters from 2978 reflections
*c* = 16.319 (3) Å	θ = 2.3–27.5º
α = 97.42 (3)º	µ = 0.38 mm^−^^1^
β = 99.07 (3)º	*T* = 113 (2) K
γ = 104.12 (3)º	Slab, colourless
*V* = 894.2 (3) Å^3^	0.22 × 0.20 × 0.12 mm

### Data collection

**Table:**

Rigaku saturn diffractometer	3155 independent reflections
Radiation source: rotating anode	2709 reflections with *I* > 2σ(*I*)
Monochromator: confocal	*R*_int_ = 0.037
*T* = 113(2) K	θ_max_ = 25.0º
ω scans	θ_min_ = 2.3º
Absorption correction: multi-scan(Crystalclear; Rigaku, 2004)	*h* = −7→7
*T*_min_ = 0.922, *T*_max_ = 0.956	*k* = −11→10
9131 measured reflections	*l* = −18→19

### Refinement

**Table:**

Refinement on *F*^2^	Secondary atom site location: difference Fourier map
Least-squares matrix: full	Hydrogen site location: inferred from neighbouring sites
*R*[*F*^2^ > 2σ(*F*^2^)] = 0.033	H-atom parameters constrained
*wR*(*F*^2^) = 0.090	*w* = 1/[σ^2^(*F*_o_^2^) + (0.0556*P*)^2^ + 0.0148*P*] where *P* = (*F*_o_^2^ + 2*F*_c_^2^)/3
*S* = 1.05	(Δ/σ)_max_ = 0.002
3155 reflections	Δρ_max_ = 0.20 e Å^−^^3^
236 parameters	Δρ_min_ = −0.27 e Å^−^^3^
Primary atom site location: structure-invariant direct methods	Extinction correction: none

### Special details

**Table:**

Geometry. All e.s.d.'s (except the e.s.d. in the dihedral angle between two l.s. planes) are estimated using the full covariance matrix. The cell e.s.d.'s are taken into account individually in the estimation of e.s.d.'s in distances, angles and torsion angles; correlations between e.s.d.'s in cell parameters are only used when they are defined by crystal symmetry. An approximate (isotropic) treatment of cell e.s.d.'s is used for estimating e.s.d.'s involving l.s. planes.
Refinement. Refinement of *F*^2^ against ALL reflections. The weighted *R*-factor *wR* and goodness of fit *S* are based on *F*^2^, conventional *R*-factors *R* are based on *F*, with *F* set to zero for negative *F*^2^. The threshold expression of *F*^2^ > σ(*F*^2^) is used only for calculating *R*-factors(gt) *etc*. and is not relevant to the choice of reflections for refinement. *R*-factors based on *F*^2^ are statistically about twice as large as those based on *F*, and *R*-factors based on ALL data will be even larger.

### Fractional atomic coordinates and isotropic or equivalent isotropic displacement parameters (Å^2^)

**Table:**

	*x*	*y*	*z*	*U*_iso_*/*U*_eq_	
Cl1	1.32350 (7)	0.97267 (5)	1.10751 (3)	0.02660 (14)	
Cl2	1.68608 (7)	0.78933 (5)	1.08859 (3)	0.02684 (14)	
O1	1.53261 (19)	0.68503 (13)	0.74819 (7)	0.0286 (3)	
N1	0.9532 (2)	0.50512 (15)	0.67138 (8)	0.0198 (3)	
N2	1.1666 (2)	0.55742 (15)	0.72303 (8)	0.0206 (3)	
C1	0.4859 (3)	0.37150 (18)	0.59400 (10)	0.0215 (4)	
H1	0.5670	0.4456	0.5692	0.026*	
C2	0.2643 (3)	0.30298 (19)	0.55983 (11)	0.0239 (4)	
H2	0.1959	0.3315	0.5120	0.029*	
C3	0.1354 (3)	0.18853 (18)	0.59586 (10)	0.0215 (4)	
C4	−0.0965 (3)	0.11843 (19)	0.56347 (11)	0.0266 (4)	
H4	−0.1698	0.1478	0.5170	0.032*	
C5	−0.2152 (3)	0.0078 (2)	0.59938 (12)	0.0298 (4)	
H5	−0.3681	−0.0362	0.5775	0.036*	
C6	−0.1072 (3)	−0.03961 (19)	0.66921 (11)	0.0265 (4)	
H6	−0.1882	−0.1154	0.6930	0.032*	
C7	0.1177 (3)	0.02613 (19)	0.70205 (11)	0.0238 (4)	
H7	0.1885	−0.0064	0.7478	0.029*	
C8	0.2442 (3)	0.14301 (18)	0.66739 (10)	0.0195 (4)	
C9	0.4748 (3)	0.21678 (18)	0.70202 (10)	0.0202 (4)	
H9	0.5469	0.1879	0.7490	0.024*	
C10	0.5944 (3)	0.33057 (17)	0.66748 (10)	0.0194 (4)	
C11	0.8300 (3)	0.40644 (18)	0.70550 (10)	0.0185 (4)	
C12	0.9545 (3)	0.37641 (18)	0.78625 (10)	0.0210 (4)	
H12A	0.9740	0.2760	0.7790	0.025*	
H12B	0.8751	0.3893	0.8321	0.025*	
C13	1.1838 (3)	0.49564 (18)	0.80228 (10)	0.0205 (4)	
H13	1.3077	0.4475	0.8076	0.025*	
C14	1.2205 (3)	0.61660 (17)	0.87818 (10)	0.0188 (4)	
C15	1.4098 (3)	0.64529 (18)	0.94150 (10)	0.0198 (4)	
H15	1.5156	0.5910	0.9367	0.024*	
C16	1.4433 (3)	0.75409 (18)	1.01202 (10)	0.0197 (4)	
C17	1.2860 (3)	0.83565 (17)	1.01969 (10)	0.0204 (4)	
C18	1.0972 (3)	0.80925 (19)	0.95591 (11)	0.0245 (4)	
H18	0.9926	0.8645	0.9605	0.029*	
C19	1.0647 (3)	0.70084 (19)	0.88558 (11)	0.0243 (4)	
H19	0.9384	0.6838	0.8429	0.029*	
C20	1.3468 (3)	0.64765 (18)	0.70068 (11)	0.0220 (4)	
C21	1.3033 (3)	0.6979 (2)	0.61719 (11)	0.0283 (4)	
H21A	1.2530	0.7871	0.6245	0.042*	
H21B	1.1888	0.6204	0.5783	0.042*	
H21C	1.4403	0.7182	0.5954	0.042*	

### Atomic displacement parameters (Å^2^)

**Table:**

	*U*^11^	*U*^22^	*U*^33^	*U*^12^	*U*^13^	*U*^23^
Cl1	0.0326 (3)	0.0253 (3)	0.0227 (2)	0.01150 (19)	0.00395 (18)	0.00158 (18)
Cl2	0.0236 (2)	0.0309 (3)	0.0231 (2)	0.00903 (19)	−0.00383 (17)	0.00134 (18)
O1	0.0188 (6)	0.0310 (7)	0.0312 (7)	−0.0007 (5)	0.0034 (5)	0.0045 (6)
N1	0.0164 (7)	0.0214 (7)	0.0185 (7)	0.0017 (6)	0.0020 (6)	0.0008 (6)
N2	0.0166 (7)	0.0245 (8)	0.0178 (7)	0.0005 (6)	0.0022 (6)	0.0046 (6)
C1	0.0215 (9)	0.0208 (9)	0.0212 (9)	0.0025 (7)	0.0054 (7)	0.0044 (7)
C2	0.0237 (9)	0.0251 (10)	0.0217 (9)	0.0047 (7)	0.0029 (7)	0.0052 (7)
C3	0.0204 (9)	0.0199 (9)	0.0222 (9)	0.0031 (7)	0.0054 (7)	−0.0006 (7)
C4	0.0210 (9)	0.0267 (10)	0.0282 (9)	0.0022 (7)	0.0017 (7)	0.0033 (8)
C5	0.0190 (9)	0.0273 (10)	0.0362 (11)	−0.0026 (8)	0.0040 (8)	−0.0001 (8)
C6	0.0260 (9)	0.0194 (9)	0.0307 (10)	−0.0022 (7)	0.0109 (8)	0.0023 (8)
C7	0.0263 (9)	0.0209 (9)	0.0228 (9)	0.0029 (7)	0.0064 (7)	0.0029 (7)
C8	0.0202 (8)	0.0172 (8)	0.0198 (8)	0.0029 (7)	0.0075 (7)	−0.0015 (7)
C9	0.0219 (9)	0.0194 (9)	0.0185 (8)	0.0043 (7)	0.0041 (7)	0.0024 (7)
C10	0.0189 (8)	0.0190 (9)	0.0194 (8)	0.0040 (7)	0.0056 (7)	0.0000 (7)
C11	0.0183 (8)	0.0176 (9)	0.0200 (8)	0.0051 (7)	0.0064 (7)	0.0018 (7)
C12	0.0201 (8)	0.0205 (9)	0.0202 (8)	0.0022 (7)	0.0021 (7)	0.0038 (7)
C13	0.0179 (8)	0.0237 (9)	0.0201 (8)	0.0046 (7)	0.0034 (7)	0.0066 (7)
C14	0.0177 (8)	0.0184 (9)	0.0202 (8)	0.0029 (7)	0.0045 (7)	0.0060 (7)
C15	0.0184 (8)	0.0211 (9)	0.0228 (9)	0.0074 (7)	0.0057 (7)	0.0079 (7)
C16	0.0181 (8)	0.0212 (9)	0.0183 (8)	0.0029 (7)	0.0007 (7)	0.0067 (7)
C17	0.0257 (9)	0.0171 (9)	0.0189 (8)	0.0049 (7)	0.0063 (7)	0.0042 (7)
C18	0.0230 (9)	0.0266 (10)	0.0274 (9)	0.0124 (8)	0.0054 (7)	0.0064 (8)
C19	0.0183 (8)	0.0288 (10)	0.0246 (9)	0.0064 (7)	−0.0001 (7)	0.0057 (8)
C20	0.0196 (9)	0.0190 (9)	0.0255 (9)	0.0023 (7)	0.0056 (7)	0.0014 (7)
C21	0.0261 (10)	0.0289 (10)	0.0283 (9)	0.0004 (8)	0.0090 (8)	0.0086 (8)

### Geometric parameters (Å, °)

**Table:**

Cl1—C17	1.7391 (17)	C9—C10	1.380 (2)
Cl2—C16	1.7329 (17)	C9—H9	0.9300
O1—C20	1.228 (2)	C10—C11	1.458 (2)
N1—C11	1.292 (2)	C11—C12	1.515 (2)
N1—N2	1.3905 (18)	C12—C13	1.541 (2)
N2—C20	1.359 (2)	C12—H12A	0.9700
N2—C13	1.482 (2)	C12—H12B	0.9700
C1—C2	1.361 (2)	C13—C14	1.513 (2)
C1—C10	1.424 (2)	C13—H13	0.9800
C1—H1	0.9300	C14—C15	1.384 (2)
C2—C3	1.423 (2)	C14—C19	1.399 (2)
C2—H2	0.9300	C15—C16	1.386 (2)
C3—C4	1.412 (2)	C15—H15	0.9300
C3—C8	1.422 (2)	C16—C17	1.390 (2)
C4—C5	1.370 (2)	C17—C18	1.389 (2)
C4—H4	0.9300	C18—C19	1.383 (2)
C5—C6	1.407 (2)	C18—H18	0.9300
C5—H5	0.9300	C19—H19	0.9300
C6—C7	1.370 (2)	C20—C21	1.504 (2)
C6—H6	0.9300	C21—H21A	0.9600
C7—C8	1.419 (2)	C21—H21B	0.9600
C7—H7	0.9300	C21—H21C	0.9600
C8—C9	1.418 (2)		
			
C11—N1—N2	108.06 (13)	C11—C12—H12A	111.2
C20—N2—N1	123.49 (13)	C13—C12—H12A	111.2
C20—N2—C13	122.90 (14)	C11—C12—H12B	111.2
N1—N2—C13	113.44 (13)	C13—C12—H12B	111.2
C2—C1—C10	120.51 (16)	H12A—C12—H12B	109.1
C2—C1—H1	119.7	N2—C13—C14	111.22 (13)
C10—C1—H1	119.7	N2—C13—C12	101.19 (13)
C1—C2—C3	121.26 (16)	C14—C13—C12	114.05 (14)
C1—C2—H2	119.4	N2—C13—H13	110.0
C3—C2—H2	119.4	C14—C13—H13	110.0
C4—C3—C8	118.65 (15)	C12—C13—H13	110.0
C4—C3—C2	122.65 (16)	C15—C14—C19	119.04 (15)
C8—C3—C2	118.70 (15)	C15—C14—C13	120.14 (14)
C5—C4—C3	121.12 (17)	C19—C14—C13	120.82 (14)
C5—C4—H4	119.4	C14—C15—C16	120.68 (15)
C3—C4—H4	119.4	C14—C15—H15	119.7
C4—C5—C6	120.44 (16)	C16—C15—H15	119.7
C4—C5—H5	119.8	C15—C16—C17	120.00 (15)
C6—C5—H5	119.8	C15—C16—Cl2	119.11 (12)
C7—C6—C5	119.83 (16)	C17—C16—Cl2	120.87 (13)
C7—C6—H6	120.1	C18—C17—C16	119.75 (15)
C5—C6—H6	120.1	C18—C17—Cl1	119.24 (13)
C6—C7—C8	121.11 (16)	C16—C17—Cl1	121.01 (13)
C6—C7—H7	119.4	C19—C18—C17	120.04 (15)
C8—C7—H7	119.4	C19—C18—H18	120.0
C9—C8—C7	122.30 (16)	C17—C18—H18	120.0
C9—C8—C3	118.87 (15)	C18—C19—C14	120.47 (15)
C7—C8—C3	118.82 (15)	C18—C19—H19	119.8
C10—C9—C8	121.33 (15)	C14—C19—H19	119.8
C10—C9—H9	119.3	O1—C20—N2	119.98 (16)
C8—C9—H9	119.3	O1—C20—C21	123.45 (15)
C9—C10—C1	119.27 (15)	N2—C20—C21	116.56 (15)
C9—C10—C11	120.09 (15)	C20—C21—H21A	109.5
C1—C10—C11	120.64 (15)	C20—C21—H21B	109.5
N1—C11—C10	122.02 (15)	H21A—C21—H21B	109.5
N1—C11—C12	113.82 (14)	C20—C21—H21C	109.5
C10—C11—C12	124.16 (14)	H21A—C21—H21C	109.5
C11—C12—C13	102.71 (13)	H21B—C21—H21C	109.5
			
C11—N1—N2—C20	171.10 (14)	C10—C11—C12—C13	−174.36 (15)
C11—N1—N2—C13	−4.34 (17)	C20—N2—C13—C14	71.20 (19)
C10—C1—C2—C3	−0.4 (2)	N1—N2—C13—C14	−113.33 (15)
C1—C2—C3—C4	178.03 (16)	C20—N2—C13—C12	−167.31 (14)
C1—C2—C3—C8	−1.9 (2)	N1—N2—C13—C12	8.16 (16)
C8—C3—C4—C5	−0.5 (3)	C11—C12—C13—N2	−8.19 (14)
C2—C3—C4—C5	179.53 (16)	C11—C12—C13—C14	111.31 (15)
C3—C4—C5—C6	−0.7 (3)	N2—C13—C14—C15	−121.67 (16)
C4—C5—C6—C7	0.6 (3)	C12—C13—C14—C15	124.67 (16)
C5—C6—C7—C8	0.7 (3)	N2—C13—C14—C19	58.3 (2)
C6—C7—C8—C9	177.62 (15)	C12—C13—C14—C19	−55.3 (2)
C6—C7—C8—C3	−1.8 (2)	C19—C14—C15—C16	1.1 (2)
C4—C3—C8—C9	−177.73 (15)	C13—C14—C15—C16	−178.91 (14)
C2—C3—C8—C9	2.2 (2)	C14—C15—C16—C17	0.0 (2)
C4—C3—C8—C7	1.8 (2)	C14—C15—C16—Cl2	−178.49 (12)
C2—C3—C8—C7	−178.31 (15)	C15—C16—C17—C18	−1.0 (2)
C7—C8—C9—C10	−179.75 (15)	Cl2—C16—C17—C18	177.50 (13)
C3—C8—C9—C10	−0.3 (2)	C15—C16—C17—Cl1	179.93 (12)
C8—C9—C10—C1	−2.0 (2)	Cl2—C16—C17—Cl1	−1.6 (2)
C8—C9—C10—C11	178.74 (14)	C16—C17—C18—C19	0.8 (2)
C2—C1—C10—C9	2.3 (2)	Cl1—C17—C18—C19	179.94 (13)
C2—C1—C10—C11	−178.40 (15)	C17—C18—C19—C14	0.3 (3)
N2—N1—C11—C10	179.26 (13)	C15—C14—C19—C18	−1.2 (2)
N2—N1—C11—C12	−1.87 (18)	C13—C14—C19—C18	178.76 (14)
C9—C10—C11—N1	174.36 (14)	N1—N2—C20—O1	−177.25 (14)
C1—C10—C11—N1	−4.9 (2)	C13—N2—C20—O1	−2.2 (2)
C9—C10—C11—C12	−4.4 (2)	N1—N2—C20—C21	3.4 (2)
C1—C10—C11—C12	176.34 (14)	C13—N2—C20—C21	178.39 (14)
N1—C11—C12—C13	6.80 (17)		

### Hydrogen-bond geometry (Å, °)

**Table:**

*D*—H···*A*	*D*—H	H···*A*	*D*···*A*	*D*—H···*A*
C6—H6···O1^i^	0.93	2.58	3.501 (2)	171

Symmetry codes: (i) *x*−2, *y*−1, *z*.

## References

[bb1] Lu, Z.-K., Li, S. & Huang, P.-M. (2006). *Acta Cryst.* E**62**, o5830–o5831.

[bb2] Rigaku (2004). *CrystalClear* Rigaku Corporation, Tokyo, Japan.

[bb3] Sheldrick, G. M. (2008). *Acta Cryst.* A**64**, 112–122. 10.1107/S0108767307043930 18156677

